# Spread of domestic animals across Neolithic western Anatolia: New stable isotope evidence from Uğurlu Höyük, the island of Gökçeada, Turkey

**DOI:** 10.1371/journal.pone.0222319

**Published:** 2019-10-10

**Authors:** Suzanne E. Pilaar Birch, Levent Atici, Burçin Erdoğu

**Affiliations:** 1 Department of Anthropology, University of Georgia, Athens, Georgia, United States of America; 2 Department of Geography, University of Georgia, Athens, Georgia, United States of America; 3 Department of Anthropology, University of Nevada, Las Vegas, Las Vegas, Nevada, United States of America; 4 Department of Archaeology, University of Akdeniz, Antalya, Turkey; University at Buffalo - The State University of New York, UNITED STATES

## Abstract

The origins of agriculture in Southwest Asia over 10,000 years ago and its subsequent spread into Europe during the Neolithic have been the focus of much archaeological research over the past several decades. Increasingly more sophisticated analytical techniques have allowed for better understanding of the complex interactions that occurred amongst humans, animals, and their environments during this transition. The Aegean Islands are critically situated where Anatolia and the mainland Greece meet, making the region pivotal for understanding the movement of the Neolithic into Europe. Located on the largest Turkish Aegean island of Gökçeada, the site of Uğurlu Höyük dates to the early Neolithic and has been the subject of ongoing excavations and research integrating a rigorous dating program with comprehensive zooarchaeological research. This paper focuses on the combination of bone collagen and tooth enamel stable isotope data with existing archaeological data to develop a fine-resolution picture of the spread of the Neolithic, particularly the importation and management of domestic fauna on Gökçeada, with broader relevance for understanding Aegean-Anatolian interactions. The stable isotope values from the fauna at Uğurlu have been used for both diachronic intrasite analyses and intersite comparisons between contemporaneous mainland sites. Integrating stable isotope and zooarchaeological datasets makes Uğurlu one of the first island sites to provide a comprehensive understanding of the geographic origin of Neolithic livestock populations and the timing of their spread from Anatolia into Europe during the process of Neolithization.

## Introduction

**Fig 1:**
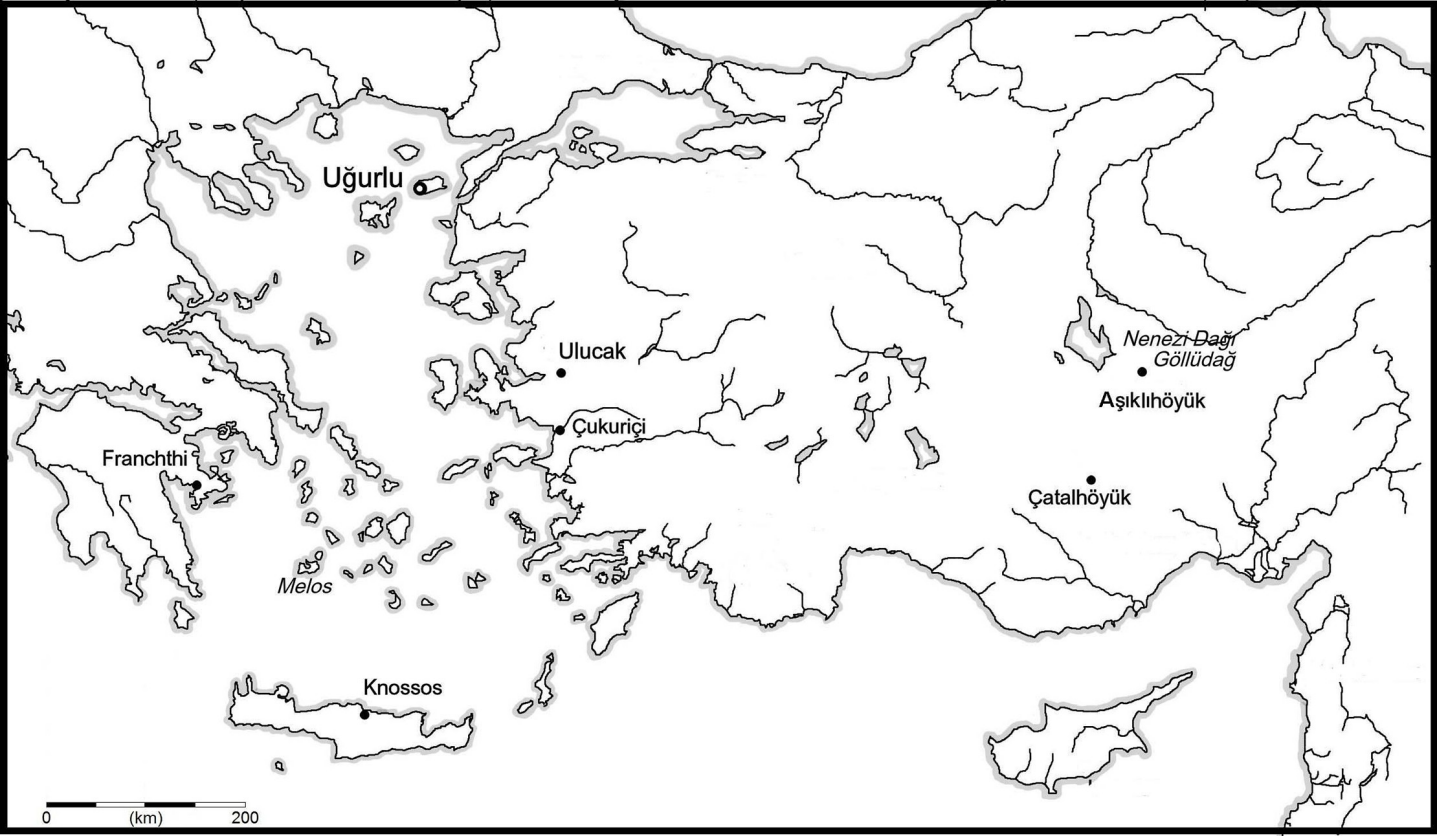
Map of the study area and environmental setting.

The current body of research surrounding the Neolithization of Europe and the spread of agricultural lifestyles from the Near East across Anatolia, the Balkans, and beyond has grown exponentially in recent years, with studies that increasingly combine emerging methodologies and techniques with extant archaeological data. It is critical that secondary types of analysis on faunal remains, such as that of stable isotopes and ancient DNA, are performed at sites and on material that has already been studied by a zooarchaeologist in order to provide a solid foundation for interpretation [1]. This paper builds on over a decade of excavations at the site of Uğurlu Höyük, Gökçeada, Turkey, directed by B. Erdoğu [2–8] and the most recent comprehensive analysis of the faunal assemblage to date, published by the authors (Atici et al.) in 2017 [9]. Here, we first incorporate the stable isotope analysis of terrestrial taxa at Uğurlu into broader anthropological questions about the spread of agriculture from approximately 6500 to 5000 cal. BC. We then situate the site and our results within a wider regional context (Fig 1) and explanatory framework. We do not set out to provide an exhaustive review of the multifaceted and complex process of the spread of domestic livestock throughout Anatolia, but rather consider the unique contribution of stable isotope data from faunal remains to our understanding of their role at Uğurlu, given its setting as both an island “endpoint” as well as a dynamic landmark on the thoroughfare of agricultural spread.

### Regional zooarchaeological context

Uğurlu Höyük is a low mound covering an area of approximately 250 × 200 m. Six main cultural phases have so far been identified and designated I-VI from the top down. In this paper, we focus on Phases V-III at Uğurlu, which correspond to the early Neolithic (6500–6000 cal BC, Phase V), late Neolithic (5900–5600 cal BC, Phase IV) and early Chalcolithic (5500–5000 cal BC, Phase III) [3]. In our previous paper [9], we discussed established relationships between Uğurlu and the island of Gökçeada with sites in western and central Anatolia, Marmara, Thrace, the Balkans, and other Aegean islands based on ceramics, technological typologies and lithic sources, and added faunal evidence to that corpus. Here, we briefly review some of these as well as other sites relevant to a discussion of faunal assemblages and their isotopic analysis. Despite the continuing growth of stable isotope analysis in archaeology in general, there are still relatively few published faunal-centered studies with large sample sizes in Turkey that date to the Neolithic. Contemporaneous sites in western Anatolia include Çukuriçi and Ulucak. At Çukuriçi, the earliest dates for the Neolithic are 6770–6480 cal BC and there are domestic sheep, goat, cattle, and some young pigs, with substantial marine input in the diet; wild animals are rare [10,11] and there is currently no stable isotope data available for comparison. At Ulucak, we see a similar subsistence economy in terms of domesticates, but very little marine input in the diet, though shells do seem to be used for decoration; wild animals are also rare [12,13]; there is published stable isotope data from caprines from both bone collagen and tooth enamel [1]. The earliest Neolithic at Ulucak dates to c. 7030–6460 cal BC; the middle Neolithic to 6650–6050 cal BC; and the late Neolithic 6030–5710 cal BC (14sea.org). In central Anatolia, there is good evidence for early domestic sheep, goat, and cows, but no pigs [9,14,15]. There, the sites of Asikli Höyük and Çatalhöyük provide two large stable isotope datasets for bone collagen and bone collagen and tooth enamel, respectively. At Aşıklı Höyük, the first half of the 8^th^ millennium BC (approximately 8,000–7,500 cal BC) was characterized by plant cultivation and had a faunal assemblage dominated by what were considered “proto-domestic” caprines (over 80%) and cows [16]. The early Neolithic at the well-known site of Çatalhöyük dates to the second half of the 8^th^ millennium BC and continues well into the late 7^th^ millennium BC, with up to 80% of the assemblage constituted by domestic sheep and goat in some levels. In the Marmara region, there is evidence for the herding of sheep, goats, and cattle by 6600 BC, and pigs by 6000 BC [13]. Contemporaneous sites include Barçın Höyük, Fikirtepe, Ilıpınar, Menteşe Höyük, and Aktopraklık. There is a faunal bone collagen stable isotope dataset from the late Neolithic Aktopraklık (6400–5600 cal.) [17,18].

Across the Aegean Sea, in the Peloponnese, the site of Franchthi famously spans thousands of years of occupation, with dates of 7028–6648 cal BC for the earliest Neolithic. Like the western Anatolian sites, there is evidence for sheep, goat, cattle, and pig; previously abundant fish and wild taxa become rare, though marine molluscs remain moderately present [19,20]. At Knossos on Crete, which was entirely depauperate of any endemic fauna prior to human settlement in the Neolithic [21–23] there is evidence for the introduction of domestic sheep, goat, cattle, pig, with limited marine input and no wild fauna. This has led to the inference of settlement by “seafaring farmers” rather than “farming seafarers” [24,25]. In contrast, though a better understanding of the chronology is needed, Maroulas on Kythnos and Cyclops on Youra both have evidence for the presence of sheep and goat, but also substantial marine assemblages, at the start of the 7^th^ millennium BC [26–28]. There are currently no large stable isotope from fauna for these sites during these time periods.

In the broader Neolithic context in Anatolia and the Aegean, then, there are certainly diverse subsistence practices. The relationship of the extant zooarchaeological data at Uğurlu in the broader context of archaeological evidence of Neolithization processes is reviewed in depth in Atici et al. 2017 [9]. Generally, while the diffusion of domesticates likely followed multiple routes, two main ‘streams’—overland and coastal—have gained favor in the literature and supported differences between distinct ‘zones’ as defined by other archaeological material, such as lithics and pottery [29, 30]; see Fig 1 in [31]. A recent review by Orton et al. (2016) reinforces the concept of two ‘streams’ of movement (one inland, one coastal) into the Balkans for sheep, goat, cattle, and pig [32]. However, as evidenced by the presence of Melian obsidian on a number of Aegean islands, movement was not unidirectional, and inter-island and inter-mainland exchange networks were nuanced. Atici et al. (2017) demonstrated that the faunal assemblages on Uğurlu represent this nuanced picture through the use of domestic and wild resources through time in the Neolithic [9]. Here we will consider the site’s relationship to contemporary Anatolian sites for which stable isotope data already exist (Ulucak, Aşıklı Höyük, and Çatalhöyük), though future comparisons with other sites may be possible pending the generation and publication of more stable isotope datasets from both bone collagen and tooth enamel.

Given this background and regional context, we investigate how stable isotope evidence may illuminate animal management practices throughout the early Neolithic at Uğurlu, the earliest Neolithic site in the eastern Aegean. In order to address this question and to aide in interpretation regarding paleoenvironmental data and regional archaeology, we first sought to directly date bone from each stratum and compare it with the established chronologies. The earliest domestic fauna must have been imported to the island. Considering the diversity of possible pathways over land and across the sea, we seek to evaluate the stable isotope data of fauna from Gökçeada in the context of other known early Neolithic sites along the route of spread of agriculture for which stable isotope data are currently available in order to shed light on the possible relationships between these regional zones. We aim to identify potential “source” populations for domesticates while recognizing that these data represent a portion of a growing body of evidence in support of a nuanced approach to the application of stable isotope techniques in reconstructing the movements of livestock in the past.

### Environmental setting and its relationship to stable isotope expectations

Currently there is a lack of detailed paleoenvironmental proxy data for the island of Gökçeada. Though Gökçeada was connected to mainland Anatolia during the Last Glacial Maximum, it became an island by the early Holocene and certainly by the Neolithic [8,33,34]. Due to its proximity to the coast (22km), there was likely always some relationship to the mainland throughout the Holocene. It is the largest (289 km^2^) and westernmost Turkish island and has a maximum elevation of 673 m, at the peak of the extinct volcano İlyas Dağ. As a result, the geology of the island is constituted primarily of late Oligocene volcanic and metamorphic rock as well as limestone and Eocene deltaic sediments [35]. There are four saltwater lagoons on the island that are used for irrigation in the modern period, along with multiple reservoirs that provide drinking water. A large river, İmroz-Büyükdere, spans 3 km and would likely have been a primary source of freshwater in the early Holocene, as well as local springs and rainwater. The vegetation is typical Mediterranean, with forests comprised of black larch, oak, and Calabrian pine at higher elevations; olive trees are ubiquitous and low lying scrub covers a majority of the landscape [36]. The site of Uğurlu is located on the southwest of the island.

According to data derived from the nearest weather station in Limnos, Greece, the weather on Gökçeada is warm and dry in the summer months of July and August (average maximum temperature 30°C, average precipitation 10mm) and cooler and wetter in the winter months of December and January (average minimum temperature 5°C, average precipitation 80mm). The average annual rainfall is approximately 500mm and the mean temperature is 15°C. In comparison, the western Anatolian coast is slightly warmer and wetter (for Izmir, an average of 17°C and 700mm/year), while the Konya Plain in Central Anatolia receives just 300mm/year and has an average mean temperature of 11°C. Given the lack of more comprehensive paleo reconstructions of temperature, rainfall, and groundcover, these data will be useful when considering the stable isotope results from fauna on Gökçeada as compared to potential “source” populations at sites located in Western and Central Anatolia, below.

Generally, increased precipitation, warmer temperatures, and higher humidity suggest more productive ecosystems and C3 vegetation, for which more negative values for both δ^13^C and δ^15^N are expected [37–39]. In contrast, more positive δ^15^N values would be expected in hot, arid environments, in individuals experiencing water stress, consuming brackish water, and consuming plants growing in manured or more saline soil [40–42]. In environments that are drier and have less precipitation, C4 plants are generally more abundant (and C3 plants may behave more like C4 plants in terms of 13C discrimination during photosynthetic uptake), leading to more positive δ^13^C values and more positive δ^15^N [39,43,44]. In turn, animal diets reflect this natural pattern; herbivores consuming local vegetation will possess a local stable isotope “signature” in their soft tissues, long since decayed, and their hard tissues, available to the archaeologist [45–49]. In particular, the protein portion of bone (collagen) can be analyzed for δ^13^C and δ^15^N, while the inorganic component of tooth enamel, hydroxyapatite (carbonate) can be analyzed for δ^13^C and δ^18^O. In temperate environments, the δ^18^O_water_ values are higher in warm temperatures and lower in cooler temperatures and are likewise related to seasonal fluctuations in rainfall amount [50,51]. The δ^18^O signature recorded in tooth enamel carbonate in mammals is related to ingested0020δ^18^O such that tooth enamel δ^18^O_carbonate_ can be used as a proxy for temperature and rainfall [48,52,53]. Tooth enamel carbonate δ^13^C values are reflective of carbon isotopic values of the whole diet with herbivore bioapatite δ^13^C values higher than the diet by 12–14‰ in ruminants [54,55]. The length of time over which a tissue forms also influences what its stable isotope signature will be; for example, in ungulates, teeth form incrementally over the first 1–2 years of life. Bones, in contrast, generally reflect the last several years of life, depending on which element is sampled. Based on the relationships between climate and environmental factors such as precipitation, humidity, aridity, temperature, and vegetation type (C3 or C4), with the isotopic values recorded in bone collagen and tooth enamel carbonate, we can distinguish relative expected ranges of values related to individuals’ zone of origin: i.e., central mainland Anatolia, the western Anatolian coast or Marmara region, and the island of Gökçeada. For example, an animal living in a relatively more arid climate such as central Anatolia may exhibit elevated δ^15^N values in comparison to an animal living in a more humid setting such as the Aegean Coast. Likewise, animals living on the island of Gökçeada and the Aegean coastal areas, which experience higher average annual rainfall than the interior, are likely to record, on average, more negative δ^18^O values in their teeth than individuals derived from central Anatolia. It is also important to keep in mind temporal scale differences in local and regional climate and environmental change; for example, evidence for increased mid-Holocene aridity in southwestern Turkey [56]. The fluctuations of regional precipitation and temperature manifest in isotope ratios, and therefore specifics of isotopic composition of local vegetation [57], with implications for the interpretation of these faunal isotope data from archaeological sites.

## Materials and methods

Field recording of faunal remains was carried out by Levent Atici (2011, 2013, and 2014) and by Levent Atici and Suzanne E. Pilaar Birch (2015) on site at the Uğurlu Höyük Excavations, directed by Burçin Erdoğu. Samples were exported to the U.S. under the permit granted by the Ministry of Culture and Tourism, Turkey, number 77366169–160.01.01, dated 13 August 2015. Pretreatment of samples was carried out in the Quaternary Isotope Paleoecology Laboratory, directed by Pilaar Birch and based at the Center for Applied Isotope Studies (CAIS), University of Georgia, USA, where all stable isotope and radiocarbon analyses were conducted.

### Radiocarbon dates

Five specimens (one canid and four sheep/goat) provided radiocarbon dates from Phases V-III. These were selected in order to strengthen the existing radiocarbon chronology and provide direct dates on bones analyzed for stable isotopes in this study. At CAIS, the samples were demineralized with cold (4°C) 1 N HCl for 24 hours, filtered, and washed with deionized water. The samples were then rinsed with 0.1M NaOH to remove humic acids, washed, and rinsed with 1N HCl to remove atmospheric CO_2_. The samples were rinsed in deionized water to pH 4 (slightly acidic) and heated at 80°C for 8 hours. The solutions were filtered through glass fiber filters to isolate the total acid insoluble fraction (“collagen”) and freeze-dried. Collagen was combusted at 575°C in evacuated and sealed Pyrex tubes in the presence of CuO to produce CO_2_. The resulting CO_2_ samples were cryogenically purified from the other reaction products and catalytically converted to graphite using the method of Vogel et al. (1984)[58]. Graphite ^14^C/^13^C ratios were measured using the 0.5 MeV accelerator mass spectrometer (AMS). The sample ratios were compared to the ratio measured from the Oxalic Acid I standard (NBS SRM 4990). The sample ^13^C/^12^C ratios were measured separately using an isotope ratio mass spectrometer (IRMS) and expressed as δ^13^C with respect to PDB, with an error of less than 0.1‰. The quoted uncalibrated date is given in radiocarbon years before 1950 (years BP), using the ^14^C half-life of 5568 years. The error is quoted as one standard deviation and reflects both statistical and experimental errors. The dates have been corrected for isotope fractionation. Dates were calibrated using OxCal v.4.3.2 and the IntCal13 calibration curve; dates are reported to 1σ.

### Collagen

Out of sixty-five samples initially selected for stable isotope analysis, fifty-nine bones produced viable collagen, including five specimens that were selected for radiocarbon dating (Table 1 and S3 Table). We chose unarticulated same-sided elements within species to avoid sampling the same individual; when multiple skeletal elements from the same context and taxon were used, determinations were made based on qualitative characteristics in which we have a high confidence. Bones sampled for isotopic analysis were derived from stratigraphically secure contexts, and these contexts are reflected in the faunal specimen number. Collagen samples were prepared using a modified Longin method [59] by demineralizing fragmented 0.5 g bone chunks in 0.5 M HCl for several days. The acid was changed every two days until the sample floated and was soft. The collagen was gelatinized by heating it in pH 3.0 water at 75°C for 48 hours. Each sample was filtered using an EZEE filter and the supernatant liquor was freeze-dried. Subsamples of freeze dried collagen powders were weighed into tin capsules and analyzed on a Costech Elemental Analyzer coupled to a Finnigan Delta IV Plus IRMS. Stable carbon is reported relative to VPDB and stable nitrogen is reported relative to AIR. Standards (n = 36) were supplied from the National Institute of Standards & Technology (NIST); for carbon, polyethylene foil (δ^13^C = – 32.15 ‰) and sucrose (δ^13^C = – 10.45 ‰) were used and for nitrogen, ammonium sulfate (δ^15^N = + 20.41 ‰) and potassium nitrate () δ^15^N = + 4.7 ‰) were used. Two internal standards (spinach, δ^15^N = – 0.54 ‰; δ^13^C = – 27.44 ‰ and protein, δ^15^N = + 8.19 ‰; δ^13^C = – 17.43 ‰) were also used. Precision was better than ± 0.15 ‰.

**Table 1:** Specimen metadata for samples analyzed for bone collagen.

Site and Year	Faunal Specimen Number	Taxon	Phase	Lab ID	Sampled Element
UZH11	P5-B.50-11	Cervid	III	UZ 83	mandible
UZH11	P5-B.50-8	OC	III	UZ 84	mandible
UZH11	P5-B.50-10	OC	III	UZ 85	mandible
UZH11	P5-B.55-8	OC	III	UZ 86	mandible
UZH11	P5-B.55-4	OC	III	UZ 87	mandible
UZH11	P5-B.50-261	OC	III	UZ 88	scapula
UZH11	P5-B.55-2	OC	III	UZ 89	scapula
UZH11	P5-B.50-251	Sus	III	UZ 90	mandible
UZH11	P5-B.51-6	Cervid	III	UZ 91	scapula
UZH11	P5-B.52-3	OC	III	UZ 92	scapula
UZH11	P5.B.51-3	Sus	III	UZ 94	metapodial
UZH11	P5-B.55-1	OC	III	UZ 95	scapula
UZH11	P5-B.51-2	OC	III	UZ 96	scapula
UZH14	P5-B.128-1	OC	III	UZ 97	scapula
UZH11	P5-B.51-5	Bos	III	UZ 98	metacarpal
UZH11	P5-B.50-263	OC	III	UZ 99	scapula
UZH11	P5-B.50-1	Bos	III	UZ 101	metacarpal
UZH11	P5-B.50-257	Cervid	III	UZ 102	scapula
UZH11	P5-B.55-5	Sus	III	UZ 103	mandible
UZH11	P5-B.52-6	Sus	III	UZ 104	maxilla
UZH11	P5-B.52-10	Bos	III	UZ 105	metatarsal
UZH14	P5-B.128-4	Cervid	III	UZ 106	antler
UZH11	P5-B.55-6	Lepus	III	UZ 107	humerus
UZH11	P5-B.52-7	Lepus	III	UZ 108	ulna
UZH11	P5-B.50-252	Canid	III	UZ 109	maxilla
UZH11	P5-B.50.9	OC	III	UZ 204	mandible
UZH14	P5-B.128.3	OC	III	UZ 205	mandible
UZH11	P5-B.106.6	OC	IV	UZ 203	mandible
UZH13	P5-B.106-32	Lepus	IV	UZ 60	pelvis
UZH13	P5-B.107-2	Lepus	IV	UZ 61	scapula
UZH14	P6-B.37-1	Bos	IV	UZ 62	pelvis
UZH14	P6-B.33-9	Bos	IV	UZ 63	ulna
UZH14	P6-B.33-4	Bos	IV	UZ 64	metacarpal
UZH14	P6-B.34-40	Bos	IV	UZ 65	metapodial
UZH13	P5-B.106-9	Cervid	IV	UZ 66	mandible
UZH13	P5-B.106-10	Cervid	IV	UZ 67	mandible
UZH14	P5-B.126-1	Cervid	IV	UZ 69	pelvis
UZH15	P6-B.44-1	Dama	IV	UZ 70	calcaneus
UZH14	P6-B.34-28	OC	IV	UZ 71	pelvis
UZH14	P6-B.34-27	OC	IV	UZ 72	pelvis
UZH14	P6-B.34-17	OC	IV	UZ 74	humerus
UZH13	P5-B.107-3	Bos	IV	UZ 75	ulna
UZH14	P6-B.34-26	OC	IV	UZ 76	pelvis
UZH13	P5-B.106-15	OC	IV	UZ 77	scapula
UZH14	P6-B.33-3	OC	IV	UZ 78	scapula
UZH15	P5-B135-1	OC	IV	UZ 79	scapula
UZH14	P6-B.37-6	OC	IV	UZ 81	humerus
UZH11	P5-B.102-12	Sus	IV	UZ 80	humerus
UZH14	P6-B.35-1	Sus	IV	UZ 82	humerus
UZH14	BB120-21-B.57-69	Bos	V	UZ 51	metacarpal
UZH10	BB22-B.11-345	Lepus	V	UZ 52	radius
UZH10	BB22-B.10-57	OC	V	UA 53	humerus
UZH10	BB22-B.10-60	OC	V	UZ 54	humerus
UZH10	BB22-B.11-158	OC	V	UZ 55	humerus
UZH14	BB120-21-B.57-14	Sus	V	UZ 56	radius
UZH10	BB22-B.10-63	OC	V	UZ 58	humerus
UZH14	BB120-21-B.57-2	Cervid	V	UZ59	mandible
UZH11	BB22-B10-25	Canid	V	UZ 200	mandible
UZH11	BB20-21-B.57.1	OC	V	UZ 201	mandible
UZH11	BB20-21-B.58.4	OC	V	UZ 202	mandible

Measured δ^13^C and δ^15^N values are provided in S1 Table.

### Enamel

Thirty-eight teeth from twenty-nine individuals (9 M2-M3 pairs) were subsampled for analysis of δ^18^O and δ^13^C in tooth enamel carbonate (Table 2). The subsamples were drilled in approximately 1mm increments, <1mm deep and 3-5mm wide, perpendicular to the vertical axis of a single cusp using a Dremel Micro and 0.5mm diameter diamond-tipped drill bit and weighed between 1-5mg each. Preparation included treatment with 2% NaOCl for 24 hours at room temperature to remove any potential organic contaminants, followed by rinsing with Millipore water. Samples were then treated with 0.1M acetic acid for four hours to remove secondary carbonates before being rinsed using Millipore water (after [60]) and placed in a desiccator. Once dry, samples were weighed into exetainers, which were then flooded with helium gas under a vacuum. The sample was then reacted with 100% phosphoric acid and the resulting CO_2_ inducted to the IRMS via gas bench. All values are reported per mil (‰) with reference to the standard Vienna Pee-Dee Belemnite (VPDB) calibrated through the standards of NIST: NBS19 (δ^13^C = + 1.95 ‰ and δ^18^O = – 2.20 ‰) and RM-8545 (δ^13^C = –46.6 ‰ and δ^18^O = – 26.41 ‰)and two internal, pure calcite standards, Fisher (δ^13^C = – 0.64 ‰ and δ^18^O = – 14.90 ‰) and A1296 (δ^13^C = + 2.56 ‰ and δ^18^O = – 0.60 ‰), with precision better than ± 0.15 ‰ for both ^18^O/^16^O and ^12^C/^13^C.

**Table 2:** Specimen metadata for samples analyzed for tooth enamel.

Site and Year	Faunal Specimen Number	Taxon	Phase	Lab ID	Tooth	n Subsamples
UZH11	P5-B.50-8	OC	III	UZ 1	LRM2	6
				UZ 2	LRM3	9
UZH11	P5-B.55-4	OC	III	UZ 3	LRM2	13
				UZ 4	LRM3	12
UZH11	P5-B.55-10	OC	III	UZ 5	LRM2	16
				UZ 6	LRM3	20
UZH11	P5-B.50-9	OC	III	UZ 7	LLM2	11
UZH11	P5-B.50-10	OC	III	UZ 8	LLM2	16
UZH11	P5-B.51-24	OC	III	UZ 9	LRM2	11
UZH11	P5-B.55-8	OC	III	UZ 10	LRM2	13
UZH11	P5-B.55-25	OC	III	UZ 11	LRM2	14
UZH14	P5-B128-3	OC	III	UZ 12	LRM2	17
UZH11	P5-B.50-253	OC	III	UZ 13	LLM3	11
UZH11	P5-B.55-9	OC	III	UZ 14	LRM3	11
UZH11	P5-B.52-9	OC	III	UZ 15	LLM3	14
UZH11	P5-B.50-11	Cervid	III	UZ 35	LLM3	7
UZH13	P5-B.106-6	OC	IV	UZ 16	LRM2	13
				UZ 17	LRM3	15
UZH13	P5-B.106-7	OC	IV	UZ 18	LLM2	17
				UZ 19	LLM3	20
UZH13	P5-B.106-11	OC	IV	UZ 20	LRM2	16
				UZ 21	LRM3	16
UZH14	P6-B.33-8	OC	IV	UZ 22	LRM2	12
UZH13	P5-B.106-8	OC	IV	UZ 23	LLM2	14
UZH13	P5-B.106-12	OC	IV	UZ 24	LLM3	20
UZH14	P6-B.33-1	Cervid	IV	UZ 36	LRM2	7
				UZ 37	LRM3	10
UZH13	P5-B.106-9	Cervid	IV	UZ 38	LLM2	6
UZH13	P5-B.106-10	Cervid	IV	UZ 39	LLM3	6
UZH14	BB120-21-B.57-1	OC	V	UZ 25	LLM2	15
				UZ 26	LLM3	8
UZH14	BB120-21-B.58-4	OC	V	UZ 27	LRM2	13
				UZ 28	LRM3	15
UZH14	BB120-21-B.57-7	OC	V	UZ 29	LLM3	18
UZH14	BB120-21-B.57-8	OC	V	UZ 30	LRM3	13
UZH14	BB120-21-B.58-3	OC	V	UZ 31	LRM3	8
UZH10	BB22-B.11-293	OC	V	UZ 32	LLM3	18
UZH10	BB120-21-B.57-2	Cervid	V	UZ 40	LLM2	5

Values for each subsample can be found in S2 Table.

## Results and discussion

### Radiocarbon dates

These dates fit well within the existing understanding of site chronology, spanning from c. 6500 cal BC to 5200 cal BC (Phases V-III) (Table 3). They contribute to the existing understanding of the duration of site occupation, provide an early date for the presence of canids at the site, and allow us to anchor our stable isotope interpretation firmly within the cultural context of the period as well as compare our results to other sites in mainland Anatolia.

**Table 3:** New radiocarbon dates on bone sampled for isotopic analysis.

Site and Year	Faunal Specimen Number	Lab ID ^14^C	Taxon	Phase	Context	^14^C age years BP	Age cal BC	Age cal BP
UZH11	P5-B.50-9	UGAMS 25381	OC	III	courtyard	6260±30	5297–5220	7246–7169
UZH14	P5-B128-3	UGAMS 25382	OC	III	plastered pit	6360±30	5367–5311	7316–7260
UZH13	P5-B.106-6	UGAMS 25380	OC	IV	floor of Building 5	6570±30	5537–5485	7486–7434
UZH14	BB120-21-B.58-4	UGAMS 25379	OC	V	floor of Building 10	7100±30	6014–5927	7963–7876
UZH10	BB22-B.10-25	UGAMS 25377	CANID	V	oldest sounding	7490±30	6424–6272	8373–8221

Dates have been calibrated using OxCal v4.3.2 and IntCal13 and are shown in BC/AD and BP, reported to 1σ.

### Collagen summary

In order to analyze the collagen results, the quality of the data was first evaluated. The accepted C/N ratio is 2.9 to 3.6 [61]. Most samples had a value of 3.4 with an overall success rate of 91% (59 out of 65 samples; see S1 Table for values). Samples that failed did not have either enough carbon or nitrogen left to be measured. Three of these were from Phase V (two *Ovis/Capra* and one *Sus*), two were from Phase IV (a cervid and *Ovis/Capra*) and one from Phase III (*Sus*). Data were found to be normally distributed using a Shapiro-Wilk test.

**Fig 2:**
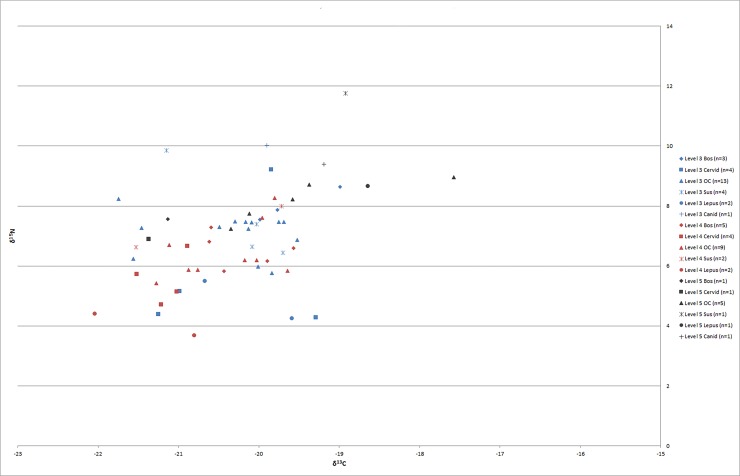
Stable isotope results of faunal collagen from Uğurlu (n = 59).

In Fig 2, shapes indicate taxa, while colors indicate the respective archaeological contexts. Though coarse, a few observations can account for the degree of variability and patterning within taxa. On average and in all contexts, deer (n = 9) and hare (n = 5) have the most negative values for both δ^13^C (-20.8‰ and -20.3‰, respectively) and δ^15^N (5.8‰ and 5.3‰). Given our expectations for these species based on their preferred forage (woodland and woodland edge) and “baseline” values for the island of Gökçeada based on physiogeographic properties, these lower values suggest that these species are likely wild and endemic rather than brought to the island from elsewhere. *Ovis/Capra* (n = 27) and *Bos* (n = 9) are indistinguishable (δ^13^C -20.2±0.8 ‰ and -20.1±0.6‰; δ^15^N 7.1±1.0‰ and 7.1±0.9‰, respectively). There is, however, more time-dependent variability in these taxa, as discussed below. Likewise, specimens of *Sus* spp. (n = 7) fall within the expected range for C3 omnivores with an average δ^13^C of -20.2‰ and δ^15^N of 8.1‰, but (in spite of small sample size) there is a shift to more negative values for both C and N in individuals through time, suggesting that these individuals may have initially arrived on the island from elsewhere and reflect “domestic” stock rather than endemic wild boar and support our earlier conclusions based on biometrically inferred size (c.f. [9]). The canid specimens (n = 2) exhibit relatively more positive δ^13^C values than the herbivores (-19.6‰) and higher δ^15^N (9.7‰) as expected for carnivores.

**Fig 3:**
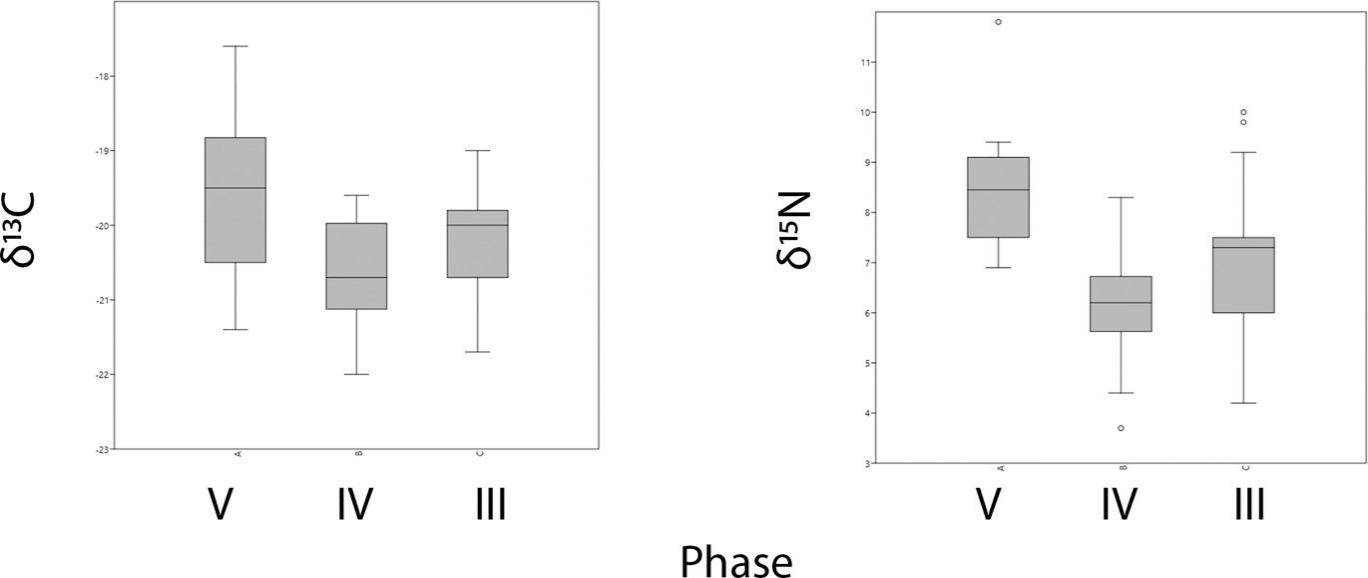
Means and variation of δ^13^C and δ^15^N values from collagen by phase.

If the data are parsed by stratum rather than by species (Fig 3), we can see that there is a notable shift in mean δ^13^C values between phases V (n = 10), IV (n = 22), and III (n = 27) using a student’s T test, with the mean δ^13^C of -19.6‰ for Phase V being significantly higher than that of IV (-20.6‰; p< 0.005). The mean δ^13^C of Phase V and III (-20.2‰; p = 0.06) were not found to be significantly different; nor are Phase IV and III (p = 0.07). An Epps-Singleton test of equal distributions found that the spread of values was not significantly different between any phase. In δ^15^N, the Phase V mean (8.5‰) is significantly higher than Phase IV (6.2‰; p<0.001) and Phase III (7.0‰; p<0.01), and IV and III also differ significantly from one another (p = 0.03). In contrast to δ^13^C, the δ^15^N values become more variable through time. The distribution of values in Phase V was significantly different from both Phase IV and III, but not between Phase IV and III. We argue that the differences in means and overall distributions of values seen here are not due to the different proportions of taxa (e.g., herbivores vs. carnivores or grazers vs. browsers) within each phase. Instead, these differences more likely reflect factors related to the environmental setting in which individual animals were raised; for example, individuals in Phase V derive from a more arid environment. Fig 3 helps to visualize differences between phases in Fig 2: in Phase V, which shows more positive δ^15^N values overall, the highest value (from a pig, an omnivore) is expressed as an outlier in the figure, while the canid (a carnivore) has an δ^15^N of 9.1‰, only marginally higher than herbivore values, from two sheep/goat individuals (8.7‰, 9.0‰) and a hare (8.7‰). These individuals also vary in their δ^13^C values, with the pig and canid notably falling between the values for the caprines and hare, supporting values influenced by individual dietary and environmental inputs independent of taxonomic status.

**Fig 4:**
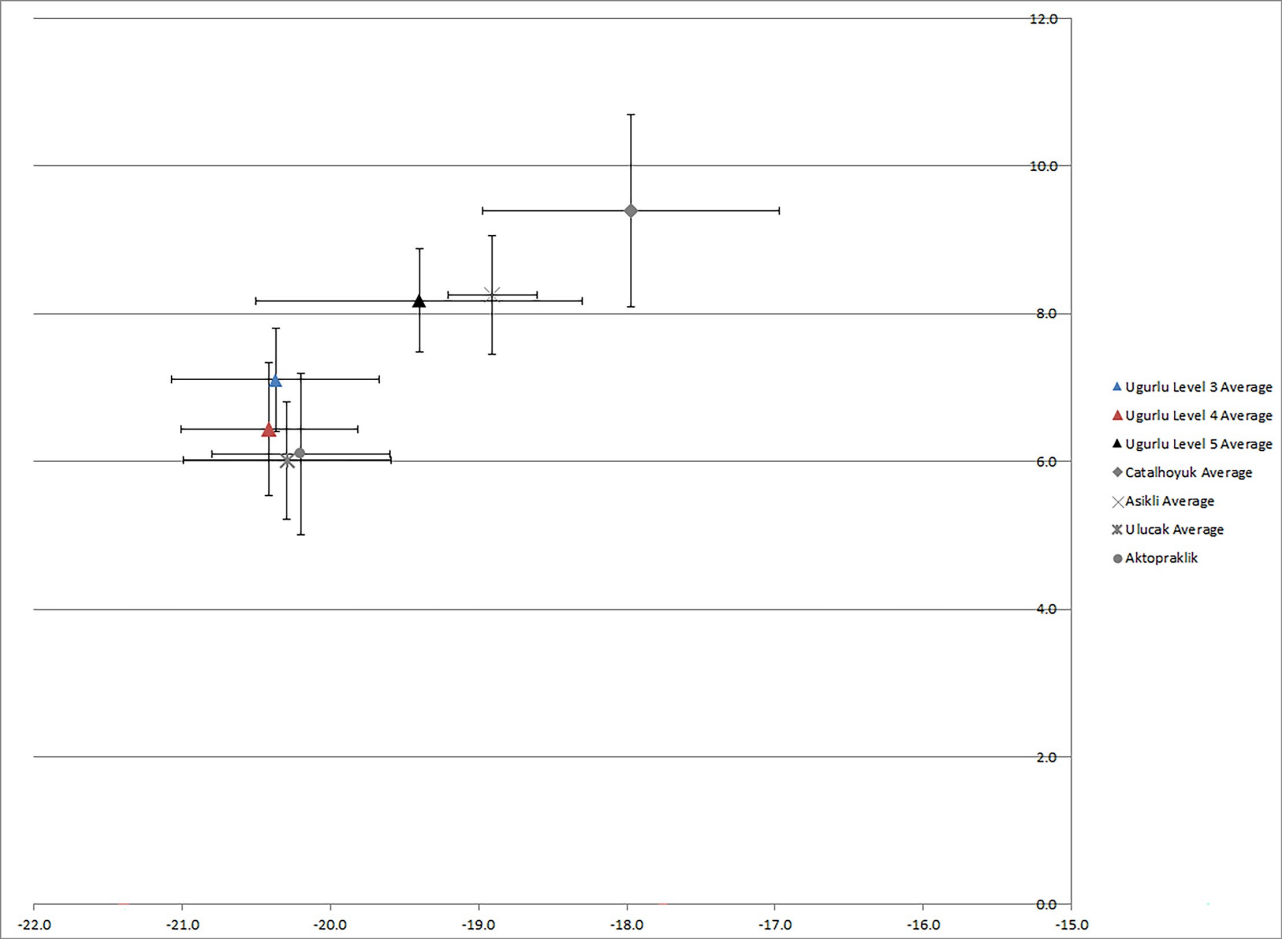
Sheep and goat bone collagen stable isotope values from Uğurlu as compared to Çatalhöyük, Aşıklı, Ulucak, and Aktopraklık.

If domesticates were imported to Uğurlu in the early Neolithic, as suggested by their high degree of inter-individual variability and significantly different mean value, where might they have been derived from? Were these island populations or were they brought in from further afield? Though the average values for sheep and goat from Uğurlu through time are fairly typical for C3 herbivores, with an overall average (n = 27) of -20.2‰ δ^13^C, 7.1‰ δ^15^N, there is a significant discrepancy in values—just within sheep/goat—between Phase V and phases IV and III (Fig 4). The average values for individuals in Phase V (n = 5) fall within the ranges of variation and closer to the mean values for contemporaneous sheep and goat individuals from Aşıklı Höyük (-18.9‰ δ^13^C, 8.1‰ δ^15^N, n = 49) and Çatalhöyük (-18.0‰ δ^13^C, 9.4 ‰ δ^15^N, n = 60) (16). In contrast, average values from Ulucak in Western Anatolia (-20.3‰ δ^13^C, 6.0‰ δ^15^N, n = 11; c.f. 1) and Aktopraklık in the Marmara (-20.2‰ δ^13^C, 6.1‰ δ^15^N, n = 13) are closer to those from later phases at Uğurlu. While not asserting that these are indeed from a Central Anatolian stock brought over to Uğurlu, it does suggest 1) an origin point that is more arid than that of Gökçeada and the Western Anatolian coast and 2) that the stock did not live longer than a few years after being brought to Gökçeada. Alternatively, regional climate may have been significantly different during the time the earliest Neolithic contexts formed such that these individuals may be reflecting temporally influenced variability rather spatial; in other words, they could derive from Gökçeada Island if it was significantly more arid overall during this time, or if there was a patchy and highly variable range of microhabitats present on the island. While we do not view these latter examples as parsimonious scenarios, it cannot be ruled out without the existence of more refined paleoenvironmental reconstructions based on local proxy data.

### Enamel summary

**Fig 5:**
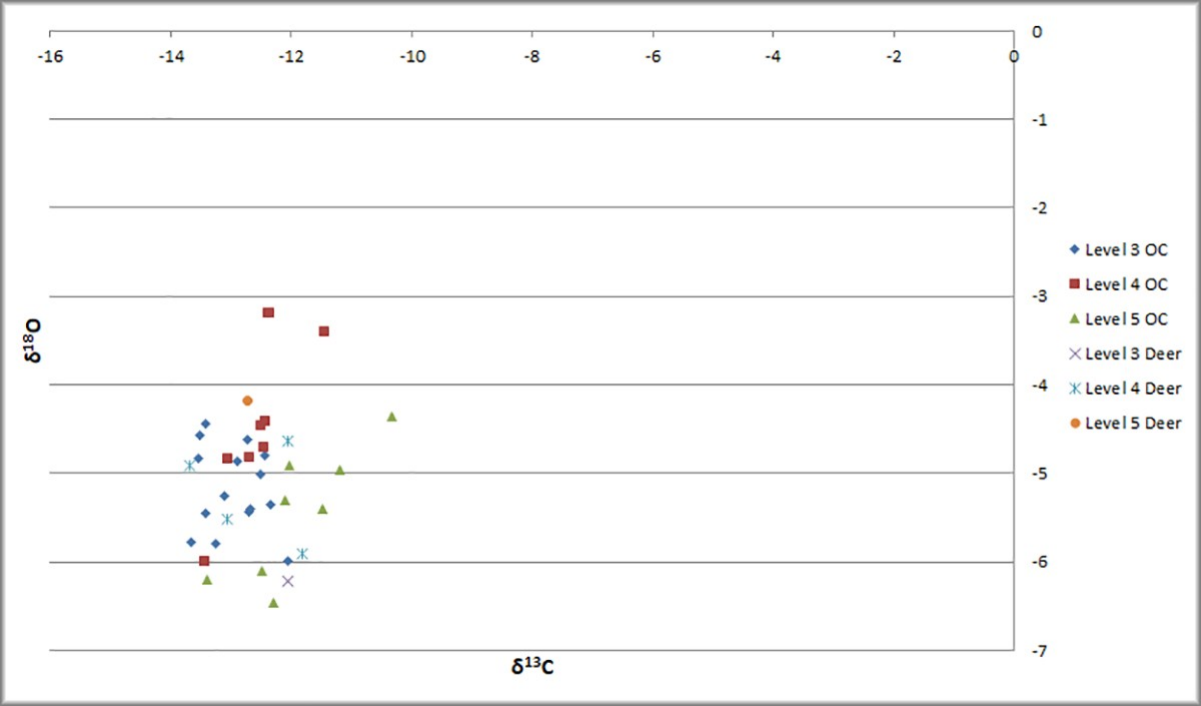
Mean tooth enamel stable isotope values for deer, sheep, and goat.

**Fig 6:**
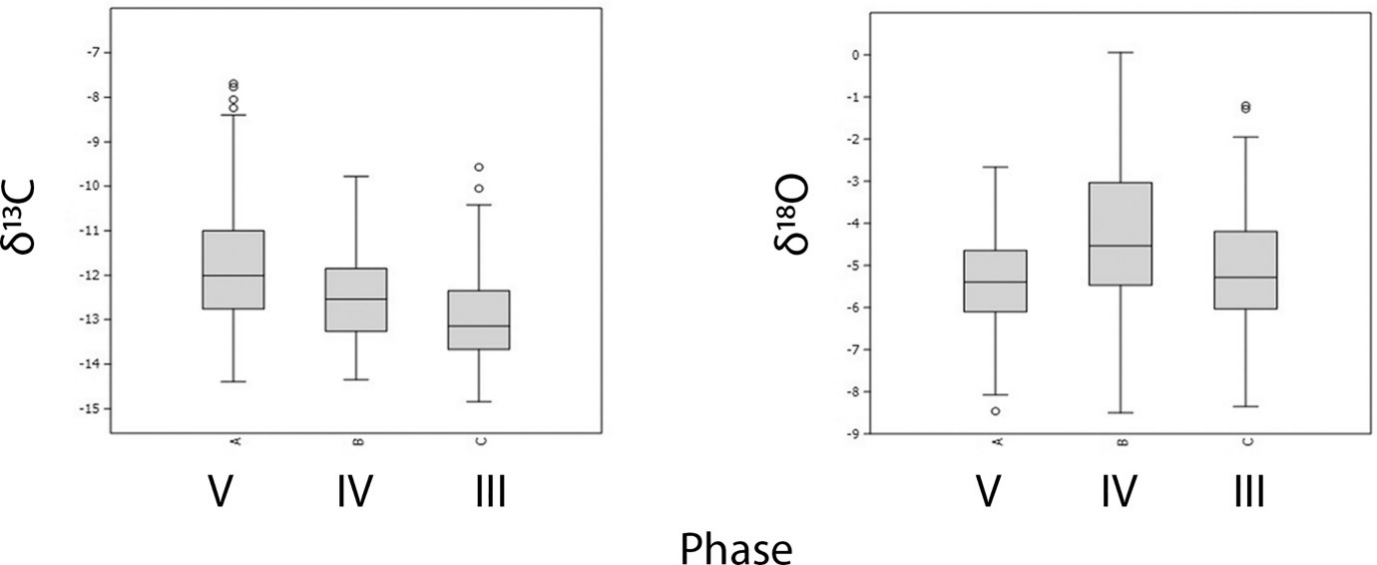
Box plots showing difference in average and variation through time at Uğurlu in sheep and goat teeth.

Stable isotope data (δ^18^O and δ^13^C) from teeth of sheep, goat, and deer were analyzed from all strata (Fig 5). The average δ^13^C for deer (n = 6 teeth) is -12.6‰, reflecting a C3 diet, whereas δ^18^O values average -5.0‰. Though the sample size is small, the values are congruent with the data derived from bone collagen in terms of suggesting habitation in a wooded setting, and the more negative δ^18^O values support the interpretation of an island/coastal location based on expectations from rainfall and temperature. There is no significant difference in the overall average δ^13^C values for sheep and goat (n = 32 teeth) (-12.6‰) nor in δ^18^O (-5.0‰), suggesting that these animals lived in a similar environment. Like bone collagen, the scenario becomes interesting when we consider variation between phases V (n = 108 values), IV (n = 135 values), and III (n = 200 values) for the sheep and goat samples (Fig 6). There is a significant trend towards more negative average δ^13^C values through time (Student’s t test; p<0.001) and, as with the collagen results, the most variation within Phase V. In particular, there are “real” outliers with more positive δ^13^C values, suggesting that these may be part of an outgroup who are derived from a different environment than those clustering with the endemic deer. However, the δ^18^O values lack this trend through time, with the average δ^18^O for Phase V at -5.4‰, Phase IV at -4.3‰, and Phase III at -5.2‰, well within the average standard deviation (1.5‰) in δ^18^O in each phase; therefore, there is no significant change in mean δ^18^O through time in sheep and goat.

**Fig 7:**
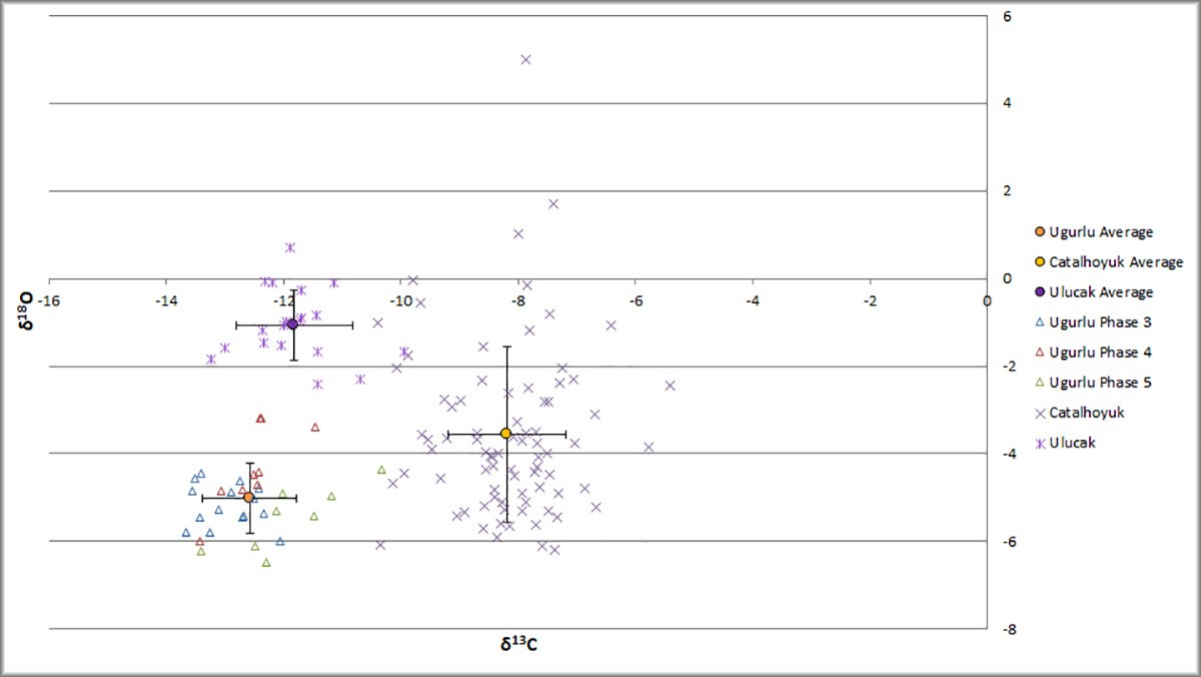
Sheep and goat tooth enamel values as compared to contemporaneous populations at Çatalhöyük and Ulucak.

As with the bone collagen, it is also possible to consider the tooth enamel data in regional context (Fig 7). The average values for sheep and goat from Uğurlu in the Neolithic are more negative in both δ^18^O and δ^13^C than comparable populations at the sites of Ulucak (n = 19) (1) and Çatalhöyük (n = 80) [62] and pers. comm. Henton 2018, Pearson 2018). The caprine “population” at Uğurlu has an overall standard deviation of 0.8‰ in both δ^13^C and δ^18^O values, as does Ulucak. The sample from Çatalhöyük has a standard deviation of 1‰ in δ^13^C and 1.9‰ in δ^18^O. Ulucak sheep and goat have an average δ^13^C of -11.8‰, within the standard deviation of Uğurlu, and suggesting, as at Uğurlu, a C3 diet in a temperate environment. In contrast, the sheep and goat values from teeth δ^13^C from Çatalhöyük have an average of -8.2‰, significantly more positive than Uğurlu and Ulucak, and likely reflective of the mixed C3/C4 diet suggested from the collagen data, and the arid interior environment of central Anatolia. The average δ^18^O at Ulucak is -1.1‰, whereas it is -3.6‰ at Çatalhöyük. This falls contrary to expectations given modern rainfall amounts, but since δ^18^O can be influenced by a combination of climate conditions (e.g. temperature, rainfall) and physiogeographic ones (e.g., source moisture) as well as biological factors, it is difficult to address this question without more detailed local paleoclimatic and paleoenvironmental information. As with the individual remains analyzed for δ^13^C and δ^15^N in collagen, some specimens from Uğurlu are relatively more similar to specimens from Çatalhöyük (Phase V) or Ulucak (Phase IV) in their δ^13^C and δ^18^O values than they are to overall average for Uğurlu, suggesting that these individuals may have been born in mainland environments and brought to the island later. They may also have been born on the island in years with anomalous rainfall, or derive from a more arid microregion on the island, but this cannot be more rigorously investigated without local, high resolution paleoclimate records. While comparison between teeth and mandibular bone from the same individual might shed further light on this interpretation, the current sample size of individuals with samples from multiple tissue types (n = 11) needs to be expanded before further analysis can be implemented.

## Conclusions

When zooarchaeological and stable isotope ecology datasets are combined, a more comprehensive and cohesive picture of Neolithic animal management on the island of Gökçeada and in western Anatolia emerges. The results of zooarchaeological analysis at Uğurlu Höyük previously published by the authors [9] corroborate the results of stable isotope ecology analysis data presented here. More specifically, osteometric data and mean sheep and goat Logarithmic Size Index (LSI) values for the Marmara and western Anatolian Neolithic sites are similar to that of Gökçeada, particularly of the earliest Neolithic or Phase V. If we turn back to our initial considerations of animal management in the earliest Neolithic and stable isotope ecology differences between phases, there is greater variation in values within the earliest Neolithic (Phase V) fauna than subsequent phases, and a significant difference in δ^15^N values. It appears likely that caprines at least are more similar to coastal ‘populations’ than central Anatolian stock, though specimens from the earliest Neolithic phase are slightly different than later periods, suggesting a founder population from the mainland and later, a local island population. Even without more detailed paleoenvironmental data zooarchaeological and stable isotope evidence independently converge to indicate that the first Neolithic inhabitants of Gökçeada may have selected their animals from the same colonizing stock, a mainland population source, that was dispersing across western Anatolia and into mainland Greece as evidenced by similar LSI values documented at Franchthi Cave (Munro and Stiner 2015), and as compared to fauna in phases IV and III at Uğurlu Höyük.

Zooarchaeological and stable isotope ecology data each manifest a different trajectory, however, during the phases IV and III—late Neolithic and early Chalcolithic—at Uğurlu Höyük. Smaller caprine body size and increasingly young male dominated caprine kill-off patterns coupled with a more caprine-dominant species trend at Chalcolithic Uğurlu Höyük hint at a specialized animal husbandry in which sheep and goats were more intensively managed through time. The decreased body size and changes in stable isotope values suggest a selective process focusing on local animal populations on the island. Fauna such as deer and hare are likely endemic, living in a more wooded area of the island, and are not consuming the same diet as domestic sheep, goat, cattle, and pig. Overall, this compliments the case for a nuanced evolution of animal resource use through time during the process of Neolithization in the region.

## Supporting information

S1 Table Stable isotope data from bone collagen samples in this study. (XLSX)

S2 Table Stable isotope data from tooth enamel subsamples in this study. (XLSX)

S3 Table Metadata for specimens sampled for multiple analyses (stable isotopes from bone collagen and tooth enamel and radiocarbon dating). (XLSX)
